# Ontogeny of Toll-Like Receptor Mediated Cytokine Responses of South African Infants throughout the First Year of Life

**DOI:** 10.1371/journal.pone.0044763

**Published:** 2012-09-13

**Authors:** Brian A. Reikie, Rozanne C. M. Adams, Candice E. Ruck, Kevin Ho, Aleksandra Leligdowicz, Santoshan Pillay, Shalena Naidoo, Edgardo S. Fortuno, Corena de Beer, Wolfgang Preiser, Mark F. Cotton, David P. Speert, Monika Esser, Tobias R. Kollmann

**Affiliations:** 1 Division of Infectious and Immunological Diseases, Department of Pediatrics, Centre for Understanding and Preventing Infections in Children, University of British Columbia, Vancouver, British Colombia, Canada; 2 Immunology Unit, Division of Medical Microbiology, Department of Pathology, NHLS and Stellenbosch University, Cape Town, South Africa; 3 Division of Medical Virology, Department of Pathology, NHLS and Stellenbosch University, Cape Town, South Africa; 4 Department of Paediatrics and Child Health, Tygerberg Children's Hospital and Stellenbosch University, Cape Town, South Africa; 5 Faculty of Medicine, University of Calgary, Alberta, Canada; Statens Serum Institute, Denmark

## Abstract

The first year of life represents a time of marked susceptibility to infections; this is particularly true for regions in sub-Saharan Africa. As innate immunity directs the adaptive immune response, the observed increased risk for infection as well as a suboptimal response to vaccination in early life may be due to less effective innate immune function. In this study, we followed a longitudinal cohort of infants born and raised in South Africa over the first year of life, employing the most comprehensive analysis of innate immune response to stimulation published to date. Our findings reveal rapid changes in innate immune development over the first year of life. This is the first report depicting dramatic differences in innate immune ontogeny between different populations in the world, with important implications for global vaccination strategies.

## Introduction

The first year of life represents a time of marked susceptibility to infections and sub-optimal response to many vaccines [1], [2], [3]. It also is a period of important developmental changes in the immune system [1], [2], [3], [4]. As innate immunity directs the adaptive immune response following pathogen exposure or vaccination, and adaptive immune responses have been shown to be largely functional already early in life [5], [6], [7], the increased risk for infectious related morbidity and mortality as well as a suboptimal response to vaccination could at least be partially due to less effective innate immune function in neonates and infants as compared to adults [4], [5], [6], [8], [9].

Antigen presenting cells (APC) represent the key link between the innate and adaptive immune system [10], [11]. The major human APCs are monocytes, classical dendritic cells (cDC) and plasmacytoid dendritic cells (pDC), as well as B cells. Stimulation of APCs with pathogen associated molecular patterns (PAMP) induces antigen presentation, expression of costimulatory molecules and secretion of cytokines by APC that direct the ensuing adaptive immune response [12]. PAMP are recognized by the APC via pattern recognition receptors (PRR), of which Toll-like receptors (TLR) are the best studied example [12]. We previously developed a stringently controlled high-throughput platform that enables robust investigation of changes in innate immunity over time [13], [14], [15]. Using this platform, we had examined the TLR response of the four major human APC in a cohort of children born and raised in North America and found that instead of lower innate immune responses to TLR stimulation in early life, strikingly different response patterns exist that dynamically change over the first 2 years of life [16], [17]. These findings were in agreement with studies of infants born and raised in other resource-rich settings [18], [19].

However, the risk of suffering from and dying of serious infection in early life is by far the greatest in sub-Saharan Africa and other resource-poor regions of the world [20]. In our current study we prospectively followed a cohort of infants born and raised in South Africa over the first year of life. Our previously established comprehensive platform was used to analyze production of innate cytokines in key functional categories utilizing both multiplex bead array and intracellular cytokine cytometric strategies [13], [14], [15]. A dynamic development of innate immunity was identified, which was unique to the population we studied, the age of the individual, as well as the stimulus employed. Given these as well as similar previous accounts [21], [22], [23], it is likely that dramatic differences in innate immune ontogeny exist between different populations.

## Results

### Striking stimulus and age-dependent differences in secreted cytokine production in response to PRR stimulation of South African infants' whole blood within the first year of life

**Figure 1:**
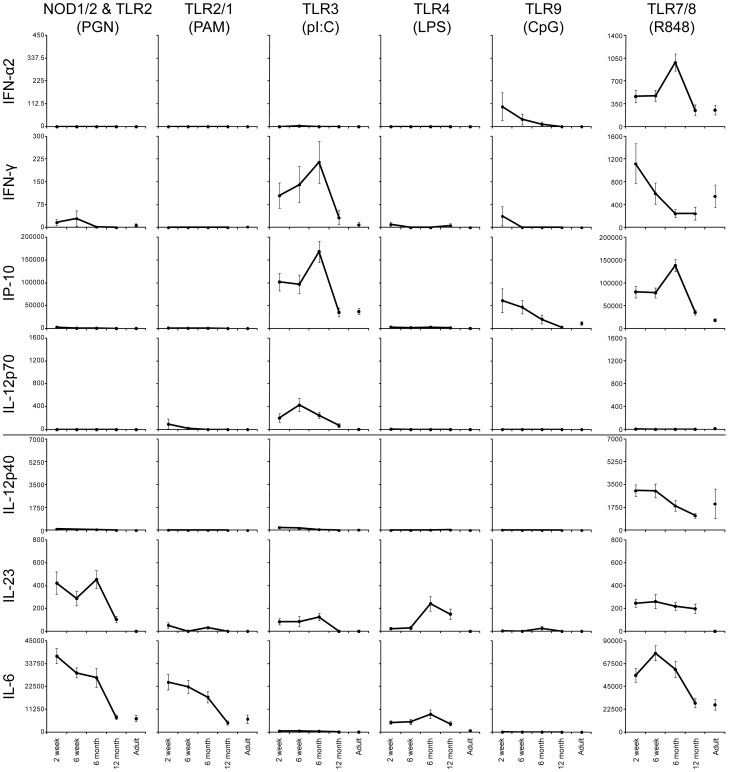
Decline in secretion of cytokines promoting either Th1 or Th17 development in South African infants' whole blood within the first year of life. Whole blood from subjects enrolled in our longitudinal cohort and sampled at 2 weeks (2 wk; n = 28), 6 weeks (6 wk; n = 26), 6 months (6 mo; n = 23) and 12 months (12 mo; n = 20) of life, and 10 adults (Ad) was stimulated with the indicated TLR ligands and cytokine secretion into the culture supernatant was measured by Luminex xMAP cytokine assay or by ELISA (IL-23 only). Mean cytokine concentration (pg/ml) is indicated on the y-axis; error bars indicate SEM. Unstimulated samples displayed cytokine production at very low levels and were subtracted from the stimulated samples. Cytokines were grouped based on their major known functions, i.e. those promoting either Th1 (IFN-α, IFN-γ and IL-12p70 as well as the IFN-γ-induced-protein IP-10), or Th17 development of CD4 T cells (IL-12p40, IL-23, and IL-6).

**Figure 2:**
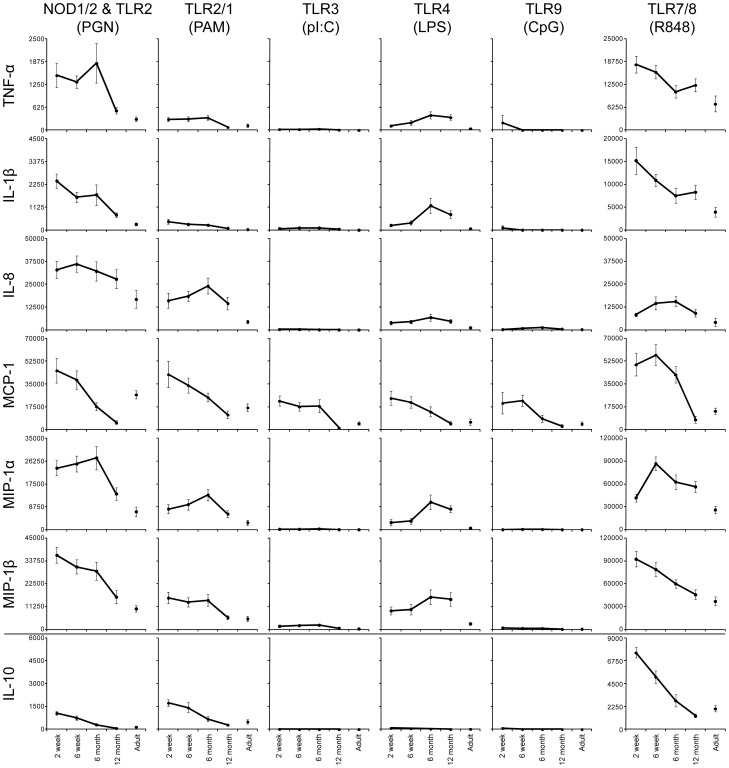
Decline of secretion of pro- as well as anti-inflammatory cytokines following PRR stimulation in South African infants' whole blood within the first year of life. The same samples analyzed in Figure 1 were also analyzed for production of cytokines considered to be general pro-inflammatory or chemoattractant (TNF-α, IL1-β, IL-8, MCP-1, MIP-1α, MIP-1β) vs. anti-inflammatory (IL-10).

Stimulation of PRRs with PAMPs allows cells expressing the relevant receptors to respond with changes in cytokine production [24]. In Figure 1 and 2 we grouped the 14 cytokines we analyzed based on their major known functions, i.e. those promoting either Th1 (IFN-α, IFN-γ and IL-12p70, as well as the IFN-γ-induced-protein, IP-10), or Th17 development of CD4 T cells (IL-12p40, IL-23 and IL-6), those considered to be general pro-inflammatory or chemoattractant (TNF-α, IL1-β, IL-8, MCP-1, MIP-1α, MIP-1β) or anti-inflammatory (IL-10).

Only TLR3, TLR7/8 or TLR9 agonists induced production of the Th1 promoting cytokines IFN-α, IFN-γ or IL-12p70 (Figure 1; Table S1). Specifically, TLR7/8 stimulation with R848 led to IFN-α secretion in younger infant samples that was significantly higher compared to the 12 month-old infant and adult samples. TLR9-induced IFN-α production followed a similar trend, from a high at 2 weeks to an adult-low by 12 months of age. TLR3-induced production of IFN-γ and IL-12p70 in infant samples at 2 weeks, 6 weeks and 6 months of age was found at levels above those of adults, with the 12-month samples approaching the adult-low level. TLR7/8-induced IFN-γ production was also highest in the 2 and 6 week infant samples, but samples from infants 6 and 12 months of age already secreted IFN-γ at or below adult samples by 6 months of age. As expected, the production of IP-10 largely followed the trends of IFN-γ, with the notable exception of a peak around 6 months of age in response to TLR7/8 stimulation.

Production of Th17 promoting cytokines (IL-23 (consisting of IL-12p40 and p19) and IL-6) occurred in response to a broader range of stimuli as compared to Th1-promoting cytokines (Figure 1; Table S1). NOD1/2 & TLR2 stimulation induced high levels of both IL-23 and IL-6 in infant samples that decreased by 12 months to an adult-low. TLR2/1 activation induced a strong IL-6 and IL-23 response that followed a similar trend, but did not induce IL-12p40 above background. TLR3 stimulation led to IL-23 production in the early infant samples that decreased to an adult low by 12 months of age yet induced no IL-6 secretion in any of the age groups tested. The TLR7/8 agonist R848 induced IL-6 secretion that dropped from an early infant high to an adult low by 12 months, but sustained higher levels of IL-23 in infant samples as compared to adult samples. TLR4 stimulation induced sustained high IL-6 production, but, contrary to all other trends, an increase in IL-23 production from a 2 and 6 week low to above adult levels at 6 and 12 months of age.

Secretion of pro-inflammatory/chemoattractant cytokines occurred in response to the widest range of stimuli (Figure 2; Table S1). For example, NOD1/2 & TLR2, TLR2/1 as well as TLR7/8 induced production of TNF-α, IL-1β, IL-8, MCP-1, MIP-1α, and MIP-1β that was high in the younger age groups, decreasing over the first year of life to reach adult-low levels around 12 months of age. TLR3 and TLR9 stimulation on the other hand only led to production of MCP-1, but followed a similar trend over time. TLR4 was again the only pathway that displayed opposite trends in comparison to other PRR. Specifically, TLR4-induced production of TNF-α, IL-1β, MIP-1α, and MIP-1β all increased from a low early in life to a high at 12 months of age; IL-8 production in response to TLR4 stimulation was overall low but higher in the infant as compared to adult samples. MCP-1 was the only cytokine produced in response to TLR4 stimulation that followed the more common trend of an early high response decreasing to an adult low by 12 months of age. Secretion of the anti-inflammatory cytokine IL-10 occurred only in response to NOD1/2 & TLR2, TLR2/1 and TLR7/8 stimulation, and generally decreased from a 2 week high over the first year of life to reach adult-low levels by 12 months of age.

When comparing the level of PRR induced cytokine secretion between each successive age group, an increase in the number of statistically significant differences was detected with increasing age for all PRR responses except for TLR4 (Table S1). Specifically, there was no difference in the production of any cytokine between 2 and 6 week old infant samples after NOD1/2 & TLR2 stimulation. Only 2 cytokines (IL-10 and MCP-1) differed significantly between 6 weeks and 6 months, but 7 cytokines (IL-10, IL-23, IL-6, MCP-1, MIP-1α, MIP-1β, and TNF-α) displayed statistically significant differences between the infants' response to NOD1/2 & TLR2 stimulation at 6 month and 12 month of age. A similar increase in the number of significant differences with advancing age was also observed following TLR2/1, TLR3, TLR7/8 and TLR9 stimulation. Again, only the response to TLR4 stimulation did not follow this trend, as cytokine secretion following TLR4 stimulation did not differ significantly between 2 and 6 week old infants, nor between 6 month and 12 month old infants, and only 2 cytokines (IL-23 and MIP-1a) differed significantly between the infants at 6 weeks and 6 months of age.

### Single cell analysis of intracellular cytokine production within the first year of life

To determine the cellular source of key cytokines detected in the culture supernatant, intracellular cytokine cytometry (ICC) was employed as previously described [14], [16], [17], [25]. Our panel of surface anchor markers allowed identification of the major APC target populations in whole blood (monocytes, B cells, cDC and pDC, Figures S1 and S2), in parallel to the expression of cytokines in each of these major APC subsets (Figure S3). A rapid rise in the fraction of B cells was detected after 2 weeks of age with a slow decline in the fraction of monocytes over the same time period; cDC and pDC populations however, remained relatively stable over the first year of life (Figures S2). Such changes in cellular composition together with changes in cellular compartments not analyzed by our flow cytometry-based approach, might affect the cytokine secretion measured in the culture supernatant described above, and thus must be considered as a variable during interpretation of results.

**Figure 3:**
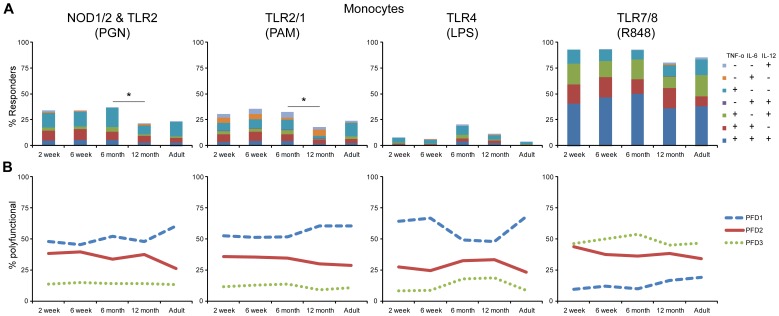
High level of monocyte cytokine response to PRR stimulation decreased between 6 and 12 months of age. Whole blood samples from neonate-infant subjects were followed longitudinally from 2 weeks (2 wk; n = 28), 6 weeks (6 wk; n = 26), 6 months (6 mo; n = 23) and 12 months (12 mo; n = 20) of life, and 10 adults (Ad) were stimulated with the indicated TLR ligands for 6 h, and intracellular cytokine levels were measured by flow cytometry for TNF-α, IL-6 and IL12/23p40 gated on monocytes. For each cell type, the total percentage of cytokine-producing cells is represented by the overall height of bar graphs; color-coded segments constituting the bar allow differentiation of cells producing various cytokine combinations. Unstimulated samples displayed cytokine production at near 0% and were subtracted from the stimulated samples. (**A**) Overall stacked bar graph; statistically significant differences in percent responders are indicated by p-value *<0.05. Cytokine profile – color combinations indicated in inset in identical order as in bar graphs from top to bottom. (**B**) Line graphs indicate polyfunctionality degree (PFD) for monocytes, summarizing the percentage of cells producing 1 (PFD1), 2 (PFD2), or 3 (PFD3) cytokines for each age group.

**Figure 4:**
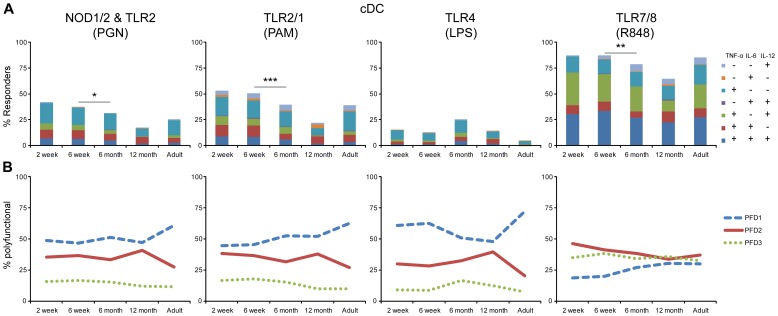
High level of cDC cytokine response to PAMPs decreased over the first year of life for all stimuli except LPS. Whole blood samples from ∼30 neonate-infant subjects were followed longitudinally from 2 weeks (2 wk; n = 28), 6 weeks (6 wk; n = 26), 6 months (6 mo; n = 23) and 12 months (12 mo; n = 20) of life, and 10 adults (Ad) were stimulated with the indicated TLR ligands for 6 h, and intracellular cytokine levels were measured by flow cytometry for TNF-α, IL-6 and IL-12/23p40 gated on cDC. For each cell type, the total percentage of cytokine-producing cells is represented by the overall height of bar graphs; color-coded segments constituting the bar allow differentiation of cells producing various cytokine combinations. Unstimulated samples displayed cytokine production at near 0% and were subtracted from the stimulated samples. (**A**) Overall stacked bar graph height; statistically significant differences in percent responders are indicated by p-value *<0.05, **<0.01, ***<0.001. Cytokine profile – color combinations indicated in inset in identical order as in bar graphs from top to bottom. (**B**) Line graphs indicate polyfunctionality degree (PFD) for cDC, summarizing the percentage of cells producing 1 (PFD1), 2 (PFD2), or 3 (PFD3) cytokines for each age group.

**Figure 5:**
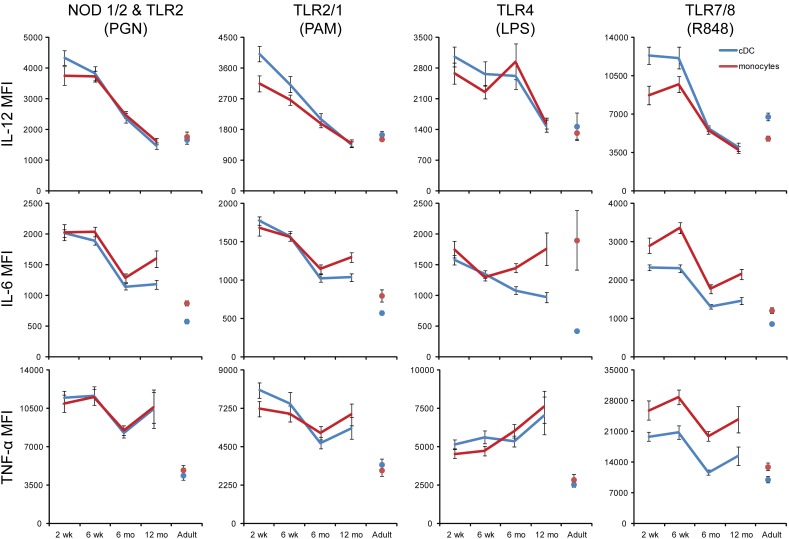
Mean fluorescence intensities (MFI) of monocyte or cDC cytokine production drastically fell over the first year of life for all cytokines except TNF-α and IL-6 in response to LPS stimulation. Whole blood samples from ∼30 neonate-infant subjects were followed longitudinally from 2 weeks (2 wk; n = 28), 6 weeks (6 wk; n = 26), 6 months (6 mo; n = 23) and 12 months (12 mo; n = 20) of life, and 10 adults (Ad) were stimulated with the indicated TLR ligands for 6 h, and intracellular cytokine levels were measured by flow cytometry for TNF-α, IL-6 and IL-12/23p40 gated on monocytes (blue line) and cDC (red line). The MFI values from all samples of each age group were averaged after first excluding the samples that had cytokine-positive percentage <4% of the APC subtype. Means for each population were derived from the FlowJo software; error bars indicate SEM.

**Figure 6:**
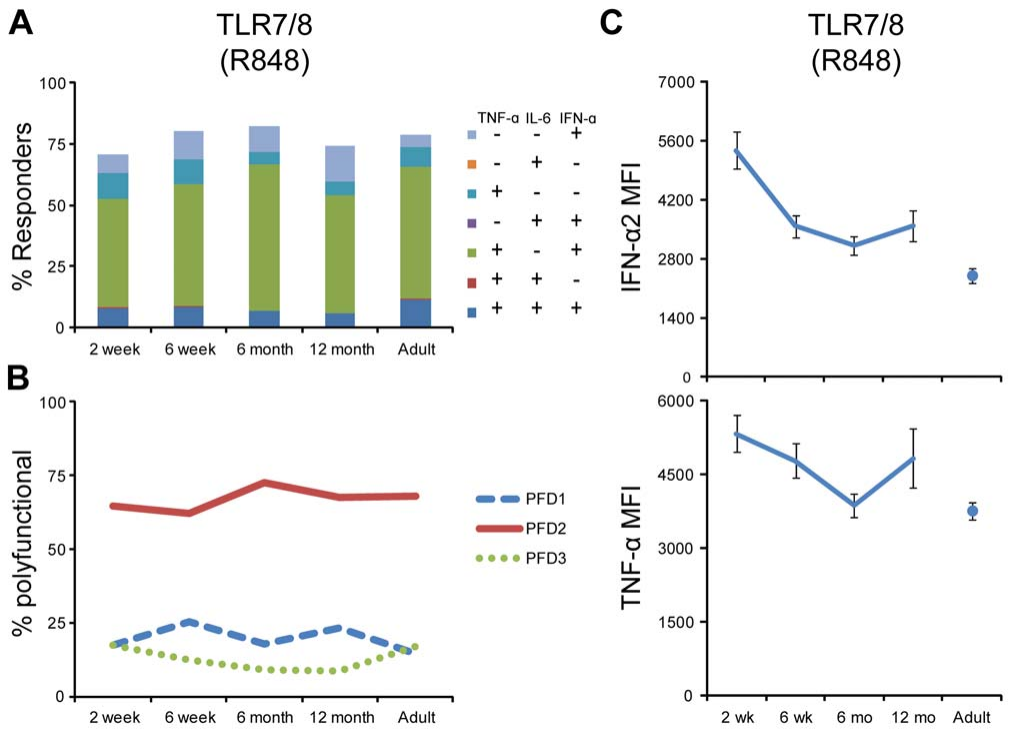
The fraction of pDC producing cytokines in response to PAMPs remained high throughout the first year of life, but the MFI for INF-α decreased. Whole blood samples from ∼30 neonate-infant subjects were followed longitudinally from 2 weeks (2 wk; n = 28), 6 weeks (6 wk; n = 26), 6 months (6 mo; n = 23) and 12 months (12 mo; n = 20) of life, and 10 adults (Ad) were stimulated with the TLR7/8 agonist R848 for 6 h, and intracellular cytokine levels were measured by flow cytometry for TNF-α, IL-6 and INF-α gated on pDC. Unstimulated samples displayed cytokine production at near 0% and were subtracted from the stimulated samples. (**A**) Overall stacked bar graph height indicates the total percentage of pDC producing one of the measured cytokines. Overall responses were broken down by cytokine expression profile and were coded by color (cytokine profile – color combinations indicated in inset in identical order as in bar graphs from top to bottom). (**B**) Line graphs indicate polyfunctionality degree (PFD) for cDC, summarizing the percentage of cells producing 1 (PFD1), 2 (PFD2), or 3 (PFD3) cytokines for each age group. (**C**) Line graphs indicate the mean fluorescent intensity (MFI) in the respective sample for INFα and TNF-α. The MFI values from all samples were averaged after first excluding the samples that have cytokine-positive percentage <4%. Means for each population are derived from the FlowJo software; error bars indicate SEM.

The polychromatic single cell flow cytometric approach enabled assessment of TNF-α, IL-6, IL-12/23p40 and IFN-α production in response to PRR stimulation of monocytes, cDC, pDC and B cells. In this study, B cells did not produce any of these cytokines under any of the conditions tested (data not shown). Monocytes and cDC did not respond to TLR3 (pI:C) or TLR9 (CpG) stimulation above background, while pDC responded to only TLR7/8 (R848) (data not shown). Therefore, analysis was focused on monocyte and cDC responses following stimulation of TLR2 & NOD1/2 (PGN), TLR1/2 stimulation (PAM), TLR4 (LPS), and TLR7/8 (R848) (Figures 3, 4 and 5), and on pDC responses, after TLR7/8 (R848) stimulation (Figure 6). TLR/NOD stimulation of monocytes and cDC elicited TNF-α, IL-6, and IL-12/23p40 responses, but not IFN-α, whereas TLR7/8 stimulation of pDC elicited TNF-α, IL-6, and IFN-α responses, but not IL-12/23p40. Therefore, data only for the 3 cytokines produced by each respective cell type is displayed. Visualization of the degree of polyfunctionality (PFD) is further augmented for each of the innate cell subsets with line graphs illustrating the proportion of cells producing either 1, 2, or 3 cytokines in response to PRR stimulation. The IL-12 family members p35, p19, p28 and EBI3 could not be reliably detected above background by ICC with currently available reagents (data not shown); only IL-12p40 could be detected as we have previously described [14], [15], [16], [17]. This precludes differentiation of IL-12p70 vs. IL-23 by ICC nor could we exclude detection of IL-12p40 homodimers by ICC.

### High level of monocyte and cDC cytokine response to PRR stimulation decreased over the first year of life for all pathways except TLR4

Monocytes and cDC displayed an overall very similar response pattern (Figure 3 and 4). For example, NOD1/2 & TLR2 and TLR2/1 both induced production of TNF-α, IL-6, or IL-12/23p40 that was statistically significantly higher in 2 and 6-week samples as compared the 6 and 12-month infant or adult samples. While the overall response level to NOD1/2 & TLR2 and TLR2/1 stimulation in monocytes was maintained for the 6-month samples, cDCs in the same 6-month samples already displayed a statistically significant decrease to near adult levels. The 12-month infant samples displayed an overall response to NOD1/2 & TLR2 and TLR2/1 stimulation that was at or near adult responses. For cDC however, the observed response at 12 months was below the adult and the other infant age-group responses. The response to TLR7/8 stimulation resulted in maximal stimulation and this high level of response was maintained throughout infancy; only cDC in 6 and 12-month samples responded to TLR7/8 stimulation at a level statistically significantly below that of the 6 week old samples. The response pattern of monocytes and cDC to TLR4 stimulation again displayed a different pattern compared to all other stimuli: while all infant samples revealed a higher overall response as compared to adult samples, the maximal response to TLR4 stimulation was detected in the 6 month samples for both monocytes and cDC; this peak at 6 months however was not significantly different as compared to either the 6 week or the 12 month samples.

Despite the similarities in response to NOD1/2 & TLR2 and TLR2/1 stimulation, we detected substantial differences in the combination of the particular cytokines produced (Figure 3 and 4). While TNF-α-only producing monocytes and cDC represented the major population of responders to both NOD1/2 & TLR2 and TLR2/1 stimulation, TLR2/1 stimulation induced a larger fraction of IL-6- or IL-12-only producing cells as compared to NOD1/2 & TLR2 stimulation. Both NOD1/2 & TLR2 and TLR2/1 stimulation also induced a substantial fraction of cells that produce both, TNF-α and IL-6 as well as monocytes and cDC producing all three cytokines. These trends were also clearly evident in the PFD line graphs for NOD1/2 & TLR2 and TLR2/1 stimulation for both monocytes and cDC. TLR7/8 stimulation, however, induced the largest fraction of monocytes and cDCs producing all three cytokines, that in cDC reached and in monocytes even surpassed the fraction of cells producing only 1 or any 2 cytokines.

The level of expression per cell (i.e. the mean fluorescent intensity, or MFI) of IL-12 in monocytes and cDC decreased in response to every stimulus from a 2 week old high to and adult-low by 12 months of age (Figure 5). While a similar trend was observed for the MFI of IL-6 in monocytes and cDC in response to NOD1/2 & TLR2, TLR2/1 and TLR7/8 stimulation, the IL-6 MFI increased again in monocytes from a 6 week low. For the TNF-α MFI in monocytes and cDC our findings suggest an overall similar level of expression per cell across all age groups in response to NOD1/2 & TLR2, TLR2/1 and TLR7/8 stimulation, and even an increase from 2 weeks to 12 months in response to TLR4 pathway activation.

### pDC cytokine production in response to PRR stimulation remained high throughout the first year of life

The fraction of pDC responding to TLR7/8 stimulation was similar in all age groups we tested, both in overall response as well as cytokine-combination specific subsets (Figure 6A). For example, the largest fraction of pDCs were positive for both TNF-α and INF-α, followed by cells positive for INF-α only. This was also reflected in the line graph displaying PFD (Figure 6B). However, while the level of expression of INF-α per cell decreased from a 2 week high to a 12 month low, the MFI for TNF-α decreased from a 2 week high to a 6 month low, only to increase again by 12 months of age (Figure 6C).

## Discussion

This is the first longitudinal cohort study analyzing human innate immune ontogeny over the first year of life in a developing nation, and complements similar themed cross-sectional studies from resource poor settings in The Gambia [21], Ecuador [23], and Papa New Guinea [22]. Several well-characterized PRR ligands were used to stimulate whole blood samples collected in the same cohort of infants at 2 and 6 weeks, 6 and 12 months of age and compared to adult samples according to a robust innate immune profiling platform. A striking decrease from an early high to an adult low by 12 months of age was detected for nearly all cytokines analyzed in response to nearly all stimuli. Specifically, cytokines promoting Th1 or Th17 development such as IFN-α, IFN-γ, IL-12p70 or IL-23 and IL-6 decreased from a high in the first half-year of life to an adult low by 12 months of age. We observed the same age-dependent trend in response to PGN (NOD1/2, TLR2), PAM (TLR2/1), pI:C (TLR3) or R848 (TLR7/8) stimulation for the proinflammatory cytokines or chemoattractants (TNF-α, IL-1β, IL-8, MCP-1, MIP-1α and MIP-1β) as well as the anti-inflammatory IL-10. Only the response to TLR4 stimulation with LPS did not follow this trend, in that production of IL-23, IL-6, TNF-α, IL-1β, MIP-1α, MIP-1β either increased from an early low to a high at 12 months of age or maintained a higher than adult production throughout the first year of life (e.g. IL-8). LPS (TLR4) induced MCP-1 production was the only response that followed the more common age-dependent decline from an early high to an adult-low by 12 months of age. Singe-cell intracellular cytokine cytometry overall confirmed the secreted cytokine pattern, in that PGN (NOD1/2, TLR2), PAM (TLR2/1) and R848 (TLR7/8) stimulation of monocytes and cDC led to production of TNF-α, IL-6 and IL-12/23p40 that decreased from an early high to a low by 12 months of age. Again, only the response of monocytes and cDC to LPS (TLR4) followed the opposite direction of the more common age-dependent trend. The percentage of pDC responding to TLR7/8 (R848) stimulation with INF-α and TNF-α expression remained relatively constant throughout infancy. The level of expression of each cytokine per cell, i.e. the mean fluorescent intensity, reinforced these findings as only expression of TNF-α per cell remained relatively constant in all cell types across the infant age groups.

The unique pattern of increasing sensitivity to TLR4 stimulation over the first year of life is supported by previous observations that cord blood was poorly responsive to LPS as compared to adults [26]. However, the underlying mechanisms that led to the divergent trends for LPS vs. other PAMPs is currently unclear. Further investigation correlating PAMP responses and susceptibility to infectious morbidity from e.g. Gram negative (via TLR4) versus viral (via e.g. TLR3) may yield valuable information.

Given the changes in cellular composition of WB over the first year of life (Figure S1), it is difficult to interpret age-associated changes of cytokine levels detected in culture supernatants. Our previous study in North America and this current study in South Africa represent the first cohort studies to attempt identifying the cellular source of cytokines detected in supernatants extending and complementing previous studies of innate immune ontogeny which only performed secreted cytokine analysis [18], [19], [21]. We had demonstrated in prior experiments, that multiplex bead array-based analysis is largely congruent with ICC, but does not always produce the exact same pattern of cytokine expression as ICC analysis [17], [27]. The comparison of intracellular and secreted cytokine production allows contextualization of cellular responses and provides a comprehensive approach to describing innate immune ontogeny. While the trends we observed in our South African cohort in innate immune responses evaluated by ICC and multiplex bead array were the same, the magnitude of the change in response with age was much less apparent by ICC than by multiplex bead array. For example, the level of TNF-α detected in culture supernatant in response to TLR2/1 stimulation decreased dramatically between 6 months and 12 months of age (Figure 2), while the number of cells that produced of TNF-α in response to PAM actually remained steady (Figure 3 and 4) and in fact increased on a per cell basis (MFI, Figure 5). This suggests that either cellular sources were involved in cytokine production that we did not assess by flow cytometry (e.g. NK, NKT, or γδ-T cells), or that cell-cell interactions and responses to secreted cytokines were inhibited by the secretion inhibitors used in the experimental set up for flow cytometry. Both of these hypotheses can be interrogated by expanding the range of target cells for ICC and a time course of addition of cytokine secretion inhibitors. We were unable to detect our target cytokines in B cells in response to PAMP stimulation, which confirms previous findings [17], but stands in contrast to data from animal studies [28], which demonstrated cytokine production from marginal zone and follicular B cells in response to TLR stimulation. This may indicate differing responses in white blood cells in peripheral blood vs. tissue resident cells or differences between humans vs. mice.

Our detailed, broadly functional as well as single-cell based analysis of early life innate immune ontogeny adds insight into human innate immune development by demonstrating dynamic changes of innate immune reactivity specific to age, stimulus and cytokine response. These data also provide the first suggestion of population-specific differences in innate immune development: Contrary to our findings presented here on innate immune ontogeny in South African infants, we had previously detected an increase rather than a decrease from birth up to 2 years of age in Th1 and pro-inflammatory cytokine production following TLR stimulation in infants born and raised in North America [17]. A recent study investigating TLR polymorphisms within a South African population, and the resulting heterogeneity of immune responsiveness to TLR stimulation [29], lends some support to our observation of population specific differences in innate immune responsiveness.

Our studies on North American infants had been conducted on peripheral blood mononuclear cells (PBMC) instead of whole blood (WB), which can lead to significant differences in responses as we have shown [15]. Furthermore, our previous studies did not include infant age groups between birth and 1 year of age, and thus are difficult to directly compare with this current study. Our previous results with North American infants were however in line with studies of European or Papa New Guinean infants that used whole blood and followed infants throughout the first year of life [18], [19], [22]. Specifically, both Nguyen et al. [18] and Belderbos et al. [19] detected an increase in LPS or CpG induced INFγ, IP-10 and IL-12p70 production over the first year of life in European infants. This stands in sharp contrast to our South African cohort, where we detected either no response or a decrease over time (Figure 1).

This striking difference between North American and European vs. South African infant innate immune ontogeny suggests that differences between resource-rich vs. resource-poor countries might play a role in development of the innate immune system. Comparing the results of our current study with South African infants to those of Burl et al. [21] who studied the TLR response of infants born and raised in The Gambia, we indeed found them to be mostly similar. For example, the trend for TNF-α responses over the first year of life following stimulation with TLR2/1, TLR4 and TLR7/8 was nearly identical to ours. However, the study by Burl et al. was cross-sectional in nature, and progressive changes in innate immunity over the first year of life could thus have been influenced by inter-individual variability [24]. A similar decline consistent with our findings and those of Burl et al. in TLR induced cytokine production in whole blood was also detected in children born and raised in Ecuador; this decline was observed between 1 and 2 years of age, with no information provided about the earlier time points [23]. However, such decline was not observed in a recent study of infants in Papa New Guinea [22]. Together, this data supports the notion that different developmental trajectories of innate immune ontogeny exist for infants from different regions of the world. Given the heterogeneity of innate immune development within a given population [29], and the many variables that can influence innate immune analysis [15], this hypothesis will have to be tested in a well-controlled, direct side-by-side comparison.

There are several possible mechanisms that might contribute to differences between European and North American infants on one side, and infants from South Africa, The Gambia, Ecuador and Papa New Guinea on the other. One possibility is related to the receipt of live vaccines around birth. For example, none of the infants in either Europe or North America received either BCG or oral polio vaccine (OPV) at birth, while children in both South Africa and The Gambia received both BCG and OPV at birth. The infants in the study from Papa New Guinea received BCG at birth, but were mixed with respect to a neonatal dose of oral polio vaccine; and infants in the study from Ecuador receive only BCG at birth. The similarities between our South African study and the Gambian study by Burl et al. or the Ecuadorian study by Teran et al. and the Papa New Guinean study by Lisciandro et al. also raise the possibility that differences in host genetics and/or differences in exposure to environmental stimuli other than vaccines might influence innate immune ontogeny. Differences in early life environmental exposure associated with atopic disease in Australian children have been shown to lead to an innate immune development more akin to the trajectories we detected in South African infants [30]. And differences in innate cytokine production have been described for the same ethnic groups living in different environment [31]. On the other hand, differences in ethnicity (and by extrapolation host genetics) are known to lead to different innate immune responses to malaria infection in children in West Africa [32], suggesting differences in host genetics between South African and e.g. North American or European infants could equally be important in our findings [33]. As such, evaluation of European-descent vs. African-descent or affluent vs. poor South African infants may generate different results. The diversity of South African demographics provide a unique opportunity to investigate the precise role of genetics vs. early life live-exposures (including vaccines) on innate immune development. Irrespective of the role of impact of live vaccination, and/or genetics vs. environment, the currently available data strongly suggest that differences in innate immune ontogeny early in life between children born and raised in different regions of the world exist. How such differences impact protection from infection or response to vaccination represents an exceptionally important issue to address.

As we had done in our previous studies in Canada [16], [17], we also included ethnically-matched adults as controls in our South African study, with the aim of providing a stable comparator for the developing innate immune ontogeny. Comparing results of WB responses following PRR stimulation between North American adults to South African adults, a strikingly lower response was noted for all stimuli and all cytokines in the South African compared to North American adults. European Caucasian adult controls similar to our Canadian adults also appear more responsive to PRR stimulation than South African adults [18], [19]. It would possibly be informative to decipher how different innate immune trajectories relate to overall immune senescence [34]. While it is tempting to speculate that the trajectory we observed in South African infants to reach adult-like low responsiveness by 12 months of age could be a general difference between resource-rich vs. resource-poor settings affecting all age groups, and possibly relate to the higher infectious morbidity and mortality across all age groups in resource-poor vs. resource-rich regions of the world, this hypothesis will need to be directly tested.

Infant morbidity and mortality from infectious disease are highest during the first year of life [35], identifying the first 12 months of life as the most critical period to induce protection from pathogens through vaccination [2]. Contrasting our findings to our previous studies in North America, others from Northern Europe, and those from Africa, South America or the Pacific Islands, it is likely that differences in innate immune ontogeny early in life exist in different populations. Based on our current findings, this may in fact apply not only to early but also to adult life. The underlying mechanisms for these striking observations are not yet known. Our data strongly argue that innate immune development is highly heterogenic across the globe, suggesting that strategies aimed at preventing infection through e.g. vaccination might benefit if tailored to the specific target population.

## Materials and Methods

### Ethics Statement

This study was specifically approved by the Research Ethics Committee of Stellenbosch University and the IRB of the University of British Columbia (Protocol H09-02064 and H11-01947). Informed written consent from the next of kin, care givers or guardians on the behalf of the minors/children participants involved in our study was obtained for all study participants.

### Prospective Birth Cohort Study Design

A prospective, longitudinal cohort study commenced in 2009 in Cape Town, South Africa, to evaluate immune function in infants early in life. Infants were recruited at birth at the Tygerberg Academic Hospital (TAH) labor ward. Mothers' HIV-negative infection status was confirmed on presentation at TAH using serological HIV testing algorithms according to South African national protocol [36]. Infants were excluded from the study if any of the following criteria were met at any of time of the study: (1) The diagnosis of a significant chronic medical condition including: immunosuppression by disease or medication; cancer; bone marrow or organ transplantation; blood product administration within the last 3 months; bleeding disorder; known congenital malformation or genetic disorder; (2) If the parent or legal guardian were unable to read and/or comprehend the consent process. Additionally, any febrile illness within the last 24 h, or brief (<1 months) immunosuppressive medication use within one month, would result in a deferral of the blood draw to a later date. Fifty-four percent of enrolled subjects were male; the mean gestational age of enrolled infants in weeks was 37.9 (37.0–38.7 CI), and the mean birth weight in grams was 2986 (2830–3142 CI). Infants were followed at 0.5, 1.5, 3, 6, and 12 months (3 month innate responses were not analyzed). We initially enrolled 29 infants and were able to analyze 28 of them at 0.5 months (one infant did not provide a blood sample), 26 at 1.5 months, 23 at 6 months and 20 at 12 months. We were unable to locate and contact the 8 infants that were lost to follow up over the first year of life. Infants received their vaccinations according to South Africa's Expanded Program for Immunization (EPI). At each visit, infant health history was obtained, receipt of immunizations on the infant immunization card verified, and physical examinations conducted by medical professionals. The median duration of exclusive breastfeeding was 12 weeks. Healthy adults, unrelated to the infants, aged 24 to 47, of equal male-female ratio and ethnic background as the infant study subjects were recruited at Stellenbosch University.

### Blood sample processing

All blood draws were performed in the hospital by a trained phlebotomist. 3–5 ml of peripheral blood was drawn via sterile venipuncture into Vacutainers containing 143 USP units of sodium-heparin (Becton Dickinson (BD) Biosciences, catalog no. 8019839) using batches we had previously confirmed to be free of innate immune activating substance in assays performed as described elsewhere [14]. Blood samples were kept at room temperature and processed within <4 hours of the blood draw as described previously [14], [15]. Infant or adult peripheral blood was mixed with 37°C pre-warmed RPMI 1640 at a ratio of 1∶1. Two hundred microliters of diluted blood was added to each well of the premade plates containing the specific PAMPs at a concentration that elicited most pronounced responses without indication of toxicity [25], [37]. Cells were stimulated with either nothing, or PGNSA (TLR2, NOD1/2; InvivoGen) at 1 µg/ml, PAM3CSK4 (TLR2/1, EMC microcollections) at 1 µg/ml; poly I:C (TLR3, Amersham) at 50 µg/ml; 0111:B4 LPS (TLR4, InvivoGen) at 10 ng/ml; R848 (TLR7/8, InvivoGen) at 10 µM; CpGA (TLR9, Coley) at 25 µg/ml.

For the ICS assays, cells were incubated for 6 h at 37°C in 5% CO2. For the TLR3 and TLR9 ligands, BFA was added after 3 h, providing optimal detection of intracellular cytokine production in response to these ligands [14]. After culture, cells were treated with a final concentration of 2 mM EDTA for 15 min at 37°C, then spun down and resuspended in 100 µl of 1× BD FACS Lysing Solution, sealed, and stored frozen at −80°C until staining. An identical set of plates was incubated in parallel for 18 h without BFA; at 18 h, these plates were spun and 100 µl of supernatant was removed and frozen at −80°C for later Luminex analysis.

### Assessment of cytokines in culture supernatant

Supernatants were thawed at room temperature, and filtered through a 1.2-µm filter plate (Millipore) into a clean 96-well plate to remove any remaining cellular debris using a multi-screen HTS vacuum manifold (Millipore). The Luminex assay was performed using the Upstate/Millipore “Flex Kit” system using the high-biotin protocol and overnight incubation at 4°C. Cytokines measured were IFN-α2, IFN-γ, IP-10, IL-12p70, IL-12p40, IL-6, TNF-α, IL-1β, IL-8, MCP-1, MIP-1α, MIP-1β, IL-10. Samples were diluted 1-to-1 (10- or 20-fold if needed to fall within the standard curve) with RPMI 1640 supplemented 10% human AB serum. Assays were read using Luminex 200 Total System (Luminex) running either the Bio-plex (Bio-Rad) or the MasterPlex (MiraiBio) softwares, and the downstream analysis was performed using Excel (Microsoft) and an in-house database. To determine the IL-23 concentration, filtered supernatants were diluted 1∶4 in diluent contained in the human IL-23 (p19/p40) ELISA kit (eBioscience), and assays were performed according to the manufacturer's specifications. Plates were read at 450 nm with 570-nm subtraction. A sigmoid logistic curve was used to generate the standard curve. The responses observed in the unstimulated (negative) controls were negligible for all subjects, and subtracted from the stimulated samples for each individual.

### Staining, acquisition, and flow cytometric analysis

Preparation of the samples for flow cytometric analysis was performed as described previously [14], [25], [37]. A detailed description of antibodies (source, clone, and dilution), machine set up, and data acquisition compliant with the recently accepted MiFlowCyt reporting standards [25], [38] can be found in the Text S1. Briefly, frozen plates were thawed and spun, and pellets were resuspended in 200 µl of BD FACS Permeabilizing Solution and incubated at room temperature for 10 min. After one wash in PBS containing 0.5% BSA and 0.1% sodium azide (PBSAN), cells were stained in a final volume 100 µl of PBSAN for 30–60 min at room temperature. After two additional washes with PBSAN, cells were resuspended in PBS containing 1% paraformaldehyde and immediately analyzed on an LSRII Flow Cytometer (BD Biosciences) set up according to published guidelines [14], [25], [39]. Compensation beads (CompBeads; BD Biosciences) were used to standardize voltage settings and used as single-stain positive and negative controls as described previously [14], [25], [40]. Frozen stock of one adult WB sample stimulated with R848 was used to set up the detectors in every run to ensure MFI of cytokine positive and negative populations remained approximately the same. A total of 500,000 events were acquired per sample. Compensation was set in FlowJo (Tree Star) and samples were analyzed compensated. Gates were set based on the fluorescence-minus-one principle [41], [42]. We positioned the unstimulated flow cytometric samples as a biological negative control, and any cells producing cytokine above the cutoff in the negative controls were subtracted from the stimulated sample responses. This has been identified as the most appropriate approach for flow cytometric analysis of stimulation experiments [42].

### Statistical analysis

Graphs were prepared using Excel (Microsoft). Statistical differences between infant pairs were analyzed using the Wilcoxon matched-pair signed rank test. To correct for multiple comparisons of the secreted cytokine analysis, we computed the Bonferroni corrected acceptable Type I error rate (alpha) as 0.05/6 stimulation conditions = 0.01 (0.0083), as these would yield false discoveries at a rate of <0.05 (for a total of 6 comparisons for each data set) [43]. Accordingly, p-values of <0.01 were considered significant. To assess polyfunctionality (i.e., the ability of an individual cell to produce one vs. more than one cytokine in response to a specific stimulus), we computed the percentage of cells in a given cytokine-combination category (e.g., there are 7 possible cytokine-combination categories for 3 cytokines in which at least one cytokine is positive (23–1 = 7)). The percentages of reactive cells that were positive for only one of the 3 cytokines were added up to give the polyfunctional degree (PFD) 1 (PFD1); and separately, the percentages of reactive cells expressing any 2 of the 3 cytokines or all 3 cytokines were computed to give PFD2 and PFD3 values, respectively. Given that values of polyfunctional subsets in flow cytometry represent qualitative composites of several categories of quantitative data, and statistical analysis would thus be of little value, the flow cytometric analysis of polyfunctional cytokine production was not subjected to statistical examination.

## Supporting Information

Figure S1 **Gating strategy for antigen-presenting cell subsets in WB.** The gating strategy to identify innate immune cell subsets was as follows: monocytes (MHCII^+^, CD14^+/high^), conventional DCs (MHCII^+^, CD14^−/low^, CD123^−^, CD11c^+^), plasmacytoid DCs (MHCII^+^, CD14^−^, CD11c^−^, CD123^+^), and B cells (MHCII^+^, CD14^−^, CD11c^−^, CD123^−^).
(TIF)

Figure S2 **Antigen-presenting cell subsets in WB.** Relative percentage of WB cell APC populations gated on mononuclear cells in samples from 2 weeks, 6 weeks, 6 months and 12 months of life, and 10 adults. Error bars indicate SEM.
(TIF)

Figure S3 **Flow Cytometric analysis of cytokine producing cell subsets in WB.** As an example to illustrate flow cytometry based cytokine assessment over the first year of life and contrasted to adult control, an overlay is used to compare the unstimulated sample (blue) with the sample stimulated (red) with the TLR7/8 ligand, R848.
(TIF)

Table S1 **Increasing number of significant differences in the level of cytokine secretion following PRR stimulation with increasing age.** Whole blood from subjects enrolled in our longitudinal cohort and sampled at 2 weeks (2 wk; n = 28), 6 weeks (6 wk; n = 26), 6 months (6 mo; n = 23) and 12 months (12 mo; n = 20) of life was stimulated with the indicated TLR ligands and cytokine secretion into the culture supernatant was measured by Luminex xMAP cytokine assay or by ELISA (IL-23 only). Differences in mean cytokine concentrations between 2 successive age groups (2 vs. 6 weeks; 6 weeks vs. 6 months; 6 vs. 12 months) were compared using the Wilcoxon matched-pair signed rank test, and corrected for multiple comparisons. A corrected p-value of less than 0.01 indicates a significant difference (highlighted in yellow).
(XLS)

Text S1 (DOCX)
