# Supplementary figures and images for: Ontogeny of Toll-Like Receptor Mediated Cytokine Responses of South African Infants throughout the First Year of Life
Source: PLoS One. 2012 Sep 13;7(9):e44763. doi: 10.1371/journal.pone.0044763 (PMC3441420; doi:10.1371/journal.pone.0044763)

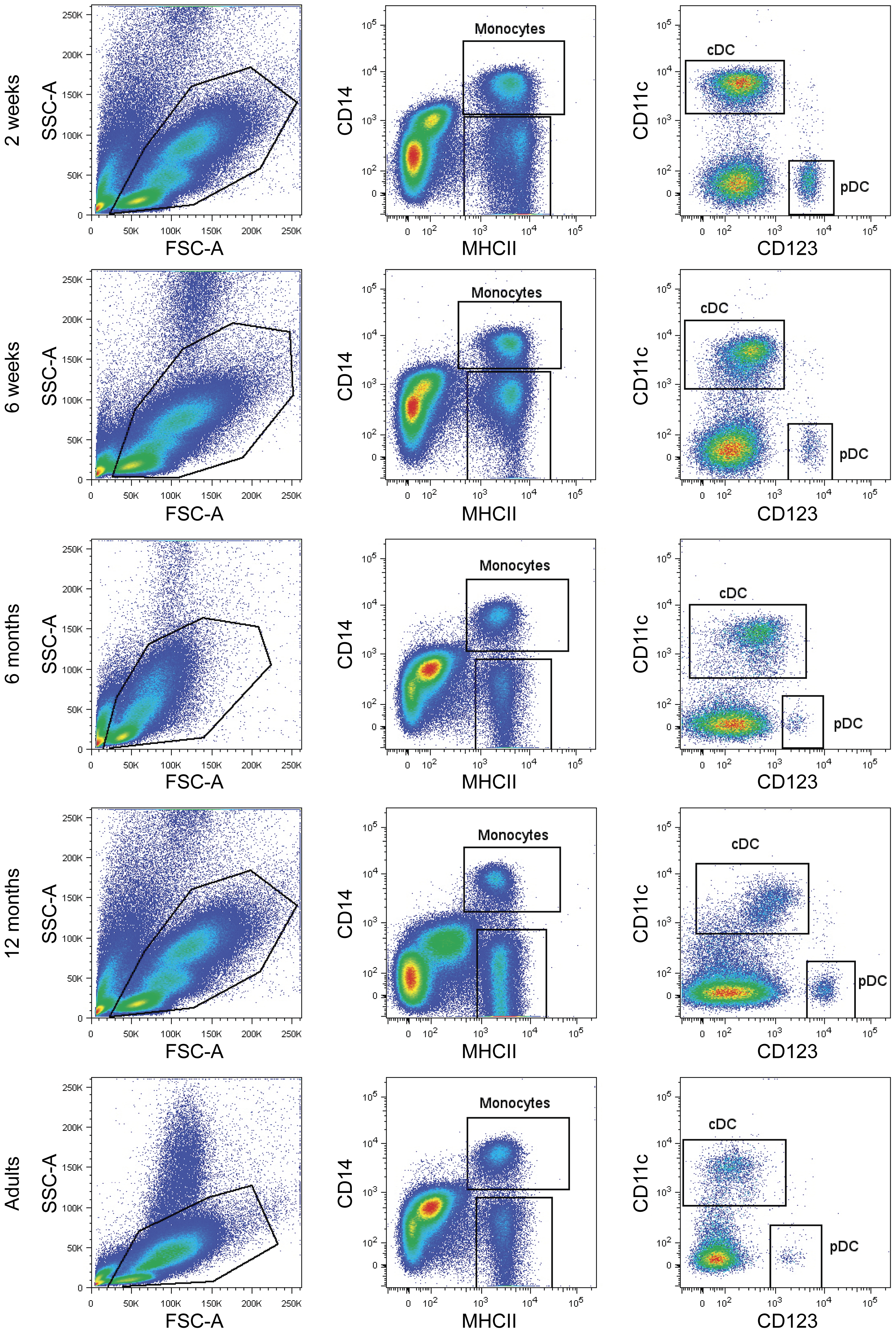

Supplement: Figure S1 — Gating strategy for antigen-presenting cell subsets in WB. The gating strategy to identify innate immune cell subsets was as follows: monocytes (MHCII+, CD14+/high), conventional DCs (MHCII+, CD14−/low, CD123−, CD11c+), plasmacytoid DCs (MHCII+, CD14−, CD11c−, CD123+), and B cells (MHCII+, CD14−, CD11c−, CD123−). (TIF) [file pone.0044763.s001.tif]

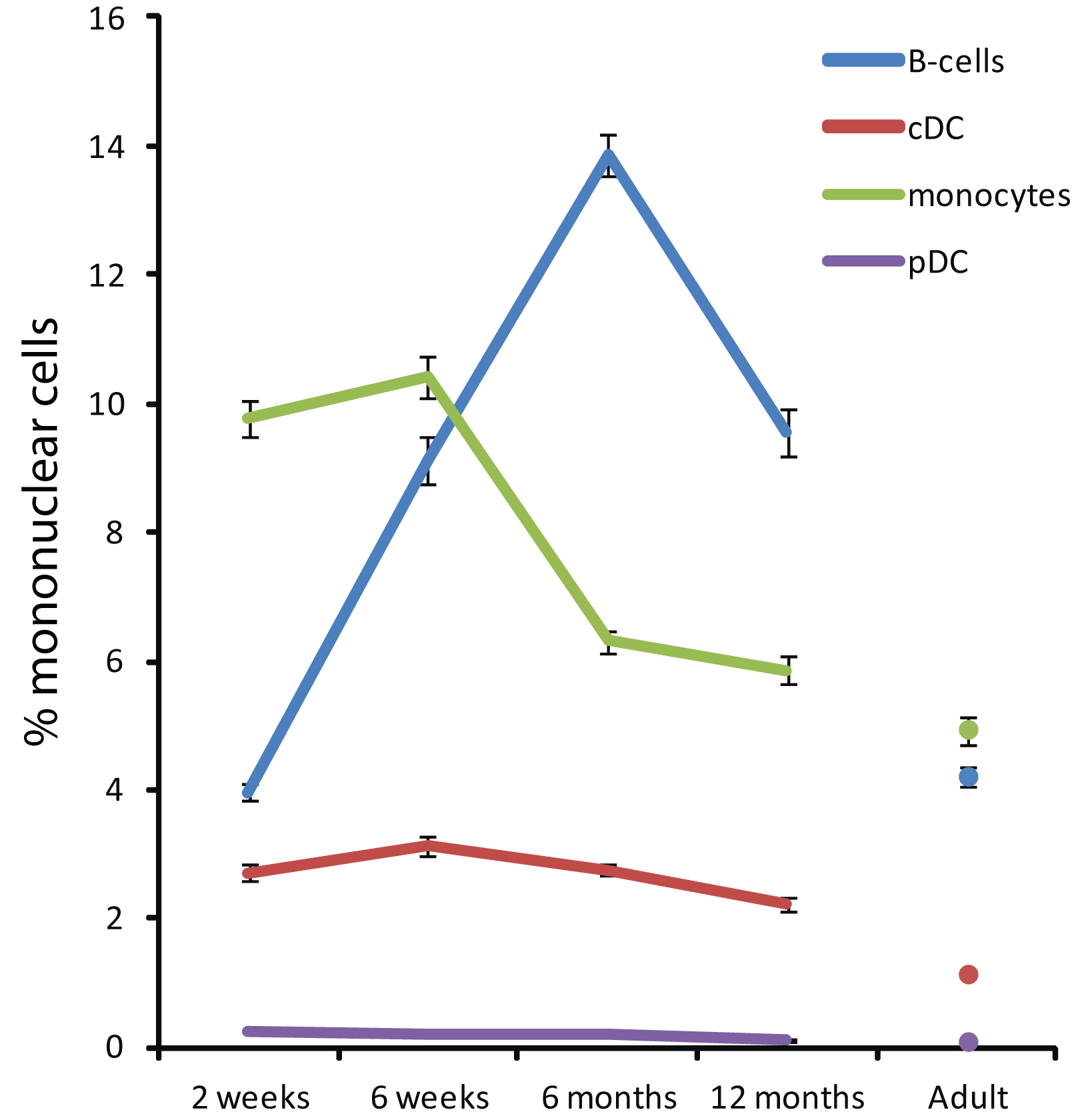

Supplement: Figure S2 — Antigen-presenting cell subsets in WB. Relative percentage of WB cell APC populations gated on mononuclear cells in samples from 2 weeks, 6 weeks, 6 months and 12 months of life, and 10 adults. Error bars indicate SEM. (TIF) [file pone.0044763.s002.tif]

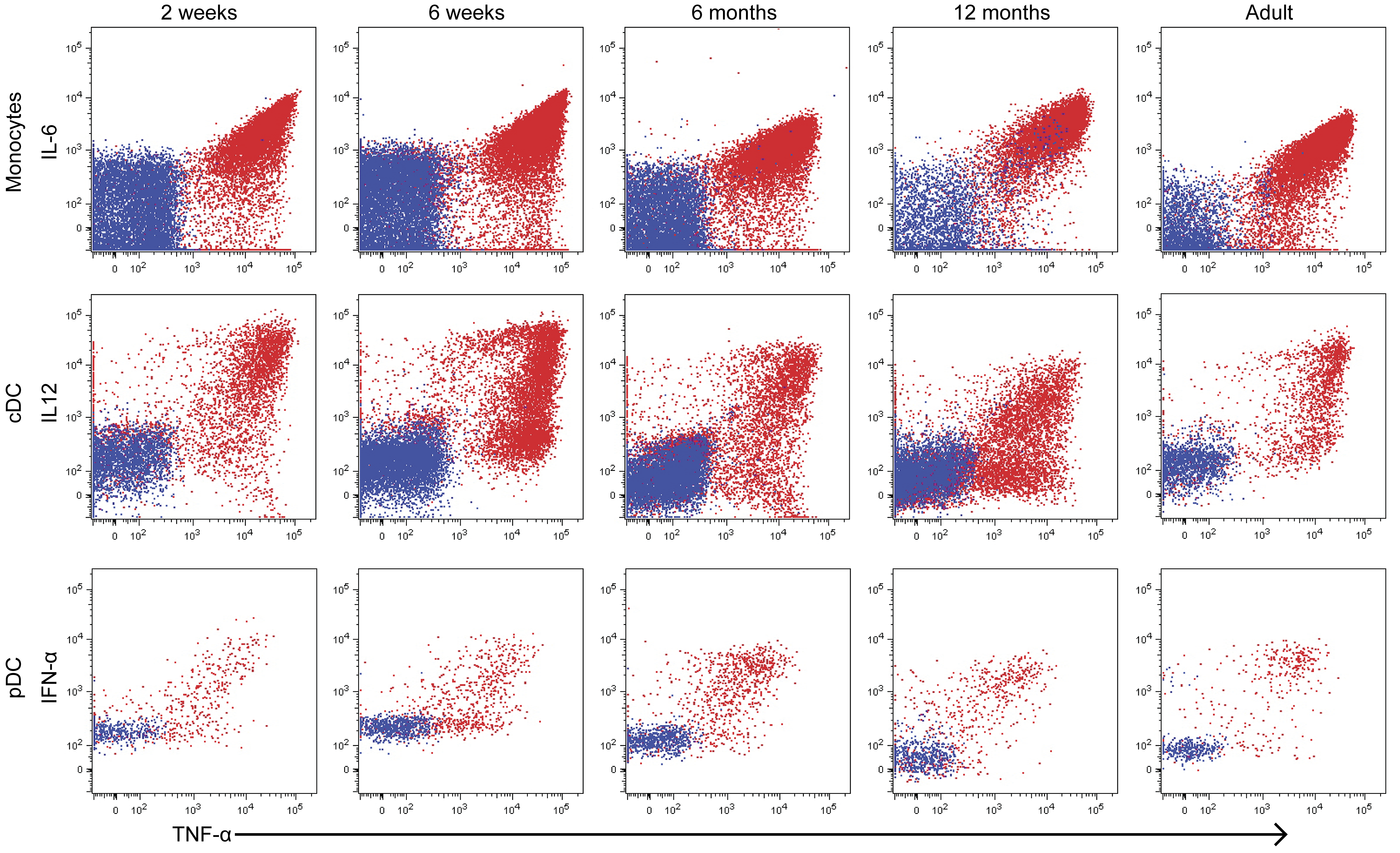

Supplement: Figure S3 — Flow Cytometric analysis of cytokine producing cell subsets in WB. As an example to illustrate flow cytometry based cytokine assessment over the first year of life and contrasted to adult control, an overlay is used to compare the unstimulated sample (blue) with the sample stimulated (red) with the TLR7/8 ligand, R848. (TIF) [file pone.0044763.s003.tif]
