# Supplementary material for: Ontogeny of Toll-Like Receptor Mediated Cytokine Responses of South African Infants throughout the First Year of Life
Source: PLoS One. 2012 Sep 13;7(9):e44763. doi: 10.1371/journal.pone.0044763 (PMC3441420; doi:10.1371/journal.pone.0044763)
Supplement: Text S1 — (DOCX) [file pone.0044763.s005.docx]

**Supplementary Data**

MIFlowCyt standard compliant information for submitted flow cytometric data.

**1. Experiment overview.**

**1.1. Purpose**: The purpose of this experiment was to track the changing responses of monocytes, B cells (by negative gating), plasmacytoid dendritic cells, and myeloid dendritic cells to TLR stimulation from 2 weeks to 12 months of life in South African infants. Comparison to adult peripheral blood was performed to give an idea as to the relationship between adult and changing early life TLR responses. This was done in whole blood to reduce the any possible processing artifact. We hypothesized that–given the changes in environment and ontogeny–that TLR responses would vary over time in the very young, and that this knowledge could form the basis of future work in rational vaccine design.

**1.2. Keywords:**

**1.3. Organization:**

**1.3.1.** Kollmann Lab, University of British Columbia

**1.3.2.** 950 W28th Ave. Vancouver, British Columbia, V5Z4H4, Room: A5-1147

**1.4. Primary Contact:**

**1.4.1.** P.I. Dr. Tobias Kollmann [tkollmann@cw.bc.ca](mailto:tkollmann@cw.bc.ca)

**1.4.2.** Research Associate. Dr. Edgardo Fortuno III [fortuno@mail.ubc.ca](mailto:fortuno@mail.ubc.ca)

**1.4.3.** Graduate Student. Mr. Brian Reikie [bareikie@ucalgary.ca](mailto:bareikie@ucalgary.ca)

**1.5. Date:** Experiments were **set up from June 2009 to June 2010 and stained from August 2010 December 2010.**

**1.6. Conclusions:**

Responses to TLR ligands change significantly from 2 weeks to 12 months of age.

**1.7. Quality Control Measures:**

Unstipulated controls were set up for each condition tested. Single stain controls were set up by staining 3 ul of Anti-Mouse Ig CompBeads (BD #552843) and 3 ul of anti-FBS negative control beads (included with BD #552843) with 3ul of each antibody used.

**2. Flow Sample/Specimen Description**

**2.1. Sample/Specimen Material**

**2.1.1. Biological Samples:**

**2.1.1.1. Biological Sample Name**

Whole blood obtained by peripheral blood venipuncture.

**2.1.1.2.Biological Sample Source:** Healthy human peripheral blood; obtained and processed within < 4h from.

**2.1.1.2.1. Biological Sample Source Organism:**

**2.1.1.2.1.1. Taxonomy:**

Kingdom Animalia Subkingdom Metazoa Phylum Chordata Subphylum Vertebrata Superclass Tetrapoda Class Mammalia Subclass Theria Infraclass Eutheria Order Primates Suborder Anthropoidea Family Hominidae Subfamily Homininae Tribe Hominini Genus Homo Subspecies sapiens

**2.1.1.2.1.2. Age:**

2 weeks to 12 months of age

**2.1.1.2.1.3. Gender:**

Male and Female

**2.1.1.2.1.4. Phenotype:**

healthy (none)

**2.1.1.2.1.5. Genotype:**

not applicable

**2.1.1.2.1.6. Treatment:**

Whole blood diluted 1:1 in RPMI.

**2.1.2. Environmental Samples:** not applicable

**2.1.3. Control Sample Description:**

Single stain controls were set up by staining 3 ul of Anti-Mouse Ig CompBeads (BD #552843) and 3 ul of anti-FBS negative control beads (included with BD #552843) with 3ul of each antibody used.

**2.1.4 Sample Treatment Description**

Cells were plated in a 96 well plate and cultured for a total of 6 hrs. Cells were stimulated with either nothing, or PGNSA (TLR2, NOD1/2, InVivogen), PAM3CSK4 (TLR2/1, EMC microcollections); poly I:C (TLR3, Amersham); 0111:B4 LPS (TLR4, InVivogen); R848 (TLR7/8, InVivogen); CpGA (TLR9, Coley). After culture, cells were treated with a final concentration of 2mM EDTA for 15 min at 37^o^C, then centrifuged @400g for 5min @22˚C and resuspended in 100ul of 1x BD FACS Lysing solution (BD 349202) for 10 minutes at room temperature before being frozen at -80 ^o^C.

**3. Fluorescence Reagent Description:**

Table 1.

|  | **Characteristic Being Measured** | **Antibody Name *Clone Name*** | **Vendor cat# *dilution used*** |
| --- | --- | --- | --- |
| **VIOLET** |  |  |  |
| **Pacific Blue** | Intracellular Protein | IL12p40/70 *(eBio: C8.6)* | eBio#577129 *1:100* |
| **RED** |  |  |  |
| **APC** | Cell Surface Protein | CD11c (5HCL3) | BD#340714 *1:50* |
| **APC-Cy7** | Intracellular Protein | IL6 *(AS12)* | BD #custom *1:100* |
| **Alexa 700** | Intracellular Protein | TNFa *(Mab11)* | BD#557996 *1:100* |
| **BLUE** |  |  |  |
| **FITC/OG** | Intracellular Protein | IFNa *(A11)* | Antigenix#MC100133 *1:100* |
| **PerCPCy5.5** | Cell Surface Protein | MHCII *TU36* | BD#custom *1:100* |
| **PE-Cy7** | Cell Surface Protein | CD14 (M5E2) | BD #557742 *1:50* |
| **PE** | Cell Surface Protein | CD123 (6H6) | eBio #121239 |

**Instrument Details:**

**3.1. Manufacturer:**

**BD Biosciences**

**3.2. Model:**

BD FACSAria Flow 3 Laser, Blue/Red/Violet serial # P22300055

**3.3. Instrument Configuration and Settings:**

All lasers, filters and mirrors were manufactured by BD Biosciences. The machine has not been altered.

**3.3.1. Light Sources:**

The light path, filters and detectors are described below in Table 2. The

lasers are listed in the order the cells pass through them. The detectors and

filters are listed in the order the light hits them, with the exception of FSC

which is measured from light that passes through the cell/bead while all the

other 488 detectors detect light that has been scattered 90 o, in the order

listed. For example, for blue laser detector A light passes through or is

reflected off of filter 1, 735 LP, then the light passes through filter 2, 780/60

BP, then it hits the PMT detector. Light that is reflected off the long pass

goes to detector B and so on. For parameters used in this experiment, it is

indicated whether Area (-A), Height (-H) or Width (-W) was used.

Abbreviations:

PMT = photomultiplier tube

PD = photodiode,

BP = band pass filter, first number is center of interval, second number is the

width of the interval.

LP = long pass filter, lets light waves through that have a longer wavelength

than the number specified. All LP filters are dichroic and reflect at an angle

of incidence at 11.25o.

Table 2.

| **Laser** | **Detector Name (Type)** | **Filter 1 (LP)** | **Filter 2 (BP)** | **Parameter detected** | **Detector voltage** | **Amplification Type** |
| --- | --- | --- | --- | --- | --- | --- |
| **Blue Laser (488 nm)** | SSC (PD) | na | 488/10 BP | SSC-A | 440 | LINEAR |
|  | 488 A (PMT) | 735 LP | 780/60 BP | PE-Cy7-A | 605 | LOG |
|  | 488 B (PMT) | 655 LP | 695/40 BP | PerCP-Cy5.5-A | 585 | LOG |
|  | 488 C (PMT) | 595 LP | 610/20 BP | PE-TexRed | na |  |
|  | 488 D (PMT) | 556 LP | 575/26 BP | PE | 498 | LOG |
|  | 488 E (PMT) | 502 LP | 530/30 BP | FITC | 480 | LOG |
| **Violet Laser (407 nm)** | 407 A (PMT) | 502 LP | 530/30 BP | Alexa-430-A | na |  |
|  | 407 B (PMT) | blank | 450/40 BP | Pacific Blue-A | 530 | LOG |
| **Red Laser (633 nm)** | 633 A (PMT) | 755 LP | 780/60 BP | APC-Cy7-A | 605 | LOG |
|  | 633 B (PMT) | 685 LP | 720/40 BP | Alexa700-A | 515 | LOG |
|  | 633 C (PMT) | blank | 660/20 BP | APC-A | 492 | LOG |

**4. Data Analysis**

**4.1. FCS Data File:**

To request raw data please contact Dr. Tobias Kollmann [tkollmann@cw.bc.ca](mailto:tkollmann@cw.bc.ca)

**4.1.1. Total Count of Events:**

Recorded within individual FCS files, as keyword $TOT, 200,000.

**4.2. Compensation Description:**

Compensation was done in FlowJo using BDCompBeads as single stain controls.


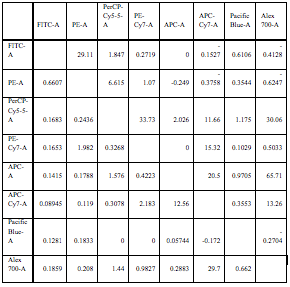
A representative compensation matrix for one of the samples is shown below

**4.3. Gating (Data Filtering) Description:**

**4.3.1. Gate Summary Information:**

**4.3.1.1. -4.3.1.3 Gate Descriptions/subpopulations/statistics:**

|  |  | **Gate Statistics (% Parent Gate)** | |
| --- | --- | --- | --- |
| **Gate Description:** | **Qualitative Description of the Subpopulation** | **Unstim (red)** | **R848 stim (blue)** |
| Live Cells | High cell density excluding lower left corner population | 61.0 | 62.9 |
| Monocytes | CD14 high, MCHII high | 8.63 | 4.61 |
| Other MHCII+ cells | MHCII high, CD14 mid to low | 6.24 | 7.02 |
| Myeloid Dendritic Cells (mDCs) | MHCII high, CD11c high, CD123 low | 41.8 | 30.7 |
| Plasmacytoid Dendritic Cells (pDCs) | MHCII high, CD11c low, CD123 high | 2.76 | 2.62 |
| Monocyte TNF+ IL-6 ­ | "Monocyte" TNFa high, IL-6 low | 0.145 | 27.5 |
| Monocyte TNF+ IL-6+ | "Monocyte" TNFa high, IL-6 high | 0.0104 | 65.6 |
| Monocyte TNF- IL-6+ | "Monocyte" TNFa low, IL-6 high | 0.28 | 0.306 |
| Monocyte TNF- IL-6­ | "Monocyte" TNFa low, IL-6 low | 99.5 | 6.65 |
| Monocyte TNF+ IL-12­ | "Monocyte" TNFa high, IL-12 low | 0.149 | 64.3 |
| Monocyte TNF+ IL-12+ | "Monocyte" TNFa high, IL-12 high | 0.00346 | 28.7 |
| Monocyte TNF- IL-12+ | "Monocyte" TNFa low, IL-12 high | 0.0588 | 0.174 |
| Monocyte TNF- IL-12­ | "Monocyte" TNFa low, IL-12 low | 99.8 | 6.78 |
| Monocyte TNF+ IFNa ­ | "Monocyte" TNFa high, IFNa low | 0.0934 | 92.1 |
| Monocyte TNF+ IFNa+ | "Monocyte" TNFa high, IFNa high | 0.0554 | 0.917 |
| Monocyte TNF- IFNa+ | "Monocyte" TNFa low, IFNa high | 0.166 | 0.535 |
| Monocyte TNF- IFNa­ | "Monocyte" TNFa low, IFNa low | 99.7 | 6.38 |
| mDC TNF+ IL-6 ­ | "mDC" TNFa high, IL-6 low | 0.194 | 40.1 |
| mDC TNF+ IL-6+ | "mDC" TNFa high, IL-6 high | 0 | 52.7 |
| mDC TNF- IL-6+ | "mDC" TNFa low, IL-6 high | 0.343 | 0.446 |
| mDC TNF- IL-6­ | "mDC" TNFa low, IL-6 low | 99.5 | 6.79 |
| mDC TNF+ IL-12­ | "mDC" TNFa high, IL-12 low | 0.194 | 43.0 |
| mDC TNF+ IL-12+ | "mDC" TNFa high, IL-12 high | 0 | 49.8 |
| mDC TNF- IL-12+ | "mDC" TNFa low, IL-12 high | 0.0915 | 2.9 |
| mDC TNF- IL-12­ | "mDC" TNFa low, IL-12 low | 99.7 | 4.34 |
| mDC TNF+ IFNa ­ | "mDC" TNFa high, IFNa low | 0.194 | 92.7 |
| mDC TNF+ IFNa+ | "mDC" TNFa high, IFNa high | 0 | 0.0297 |
| mDC TNF- IFNa+ | "mDC" TNFa low, IFNa high | 0 | 0.0149 |
| mDC TNF- IFNa­ | "mDC" TNFa low, IFNa low | 99.8 | 7.19 |
| pDC TNF+ IL-6 ­ | "pDC" TNFa high, IL-6 low | 0 | 70.0 |
| pDC TNF+ IL-6+ | "pDC" TNFa high, IL-6 high | 0 | 5.23 |
| pDC TNF- IL-6+ | "pDC" TNFa low, IL-6 high | 0 | 0 |
| pDC TNF- IL-6­ | "pDC" TNFa low, IL-6 low | 100 | 25.1 |
| pDC TNF+ IL-12­ | "pDC" TNFa high, IL-12 low | 0 | 73.7 |
| pDC TNF+ IL-12+ | "pDC" TNFa high, IL-12 high | 0 | 1.57 |
| pDC TNF- IL-12+ | "pDC" TNFa low, IL-12 high | 0 | 0.174 |
| pDC TNF- IL-12­ | "pDC" TNFa low, IL-12 low | 100 | 24.9 |
| pDC TNF+ IFNa ­ | "pDC" TNFa high, IFNa low | 0 | 5.92 |
| pDC TNF+ IFNa+ | "pDC" TNFa high, IFNa high | 0 | 69.2 |
| pDC TNF- IFNa+ | "pDC" TNFa low, IFNa high | 0 | 15.0 |
| pDC TNF- IFNa­ | "pDC" TNFa low, IFNa low | 100 | 10.1 |

**4.4. Data Transformation Description:**

Data was transformed using FlowJo’s “Define BiExponential Transformation”

function using the above mentioned compensation matrix, with an additional

negative display size set at 0.5 and Positive Decades of “log” Display set at 5.
